# A Case of Advanced Multifocal Cholangiocarcinoma With Excellent Response to Pembrolizumab

**DOI:** 10.7759/cureus.38332

**Published:** 2023-04-30

**Authors:** Tehmina Habib, Mohammad Abu-Abaa, Neel Gandhi

**Affiliations:** 1 Internal Medicine, Capital Health Regional Medical Center, Trenton, USA

**Keywords:** good response, pembrolizumab, immune checkpoint inhibitor, biliary tract malignancy, intrahepatic cholangiocarcinoma

## Abstract

The role of immune checkpoint inhibitors (ICIs) continues to receive more attention as evidence emerges of their efficacy and improved survival in patients with advanced biliary tract malignancies including cholangiocarcinoma (CCA). In line with this evidence, we describe the case of a 52-year-old male patient who presented initially in October 2019 with abdominal pain. Investigations revealed multifocal hepatic masses that proved to be CCA that was considered unresectable. Chemotherapy with cisplatin (C) and gemcitabine (G) was initiated. In January 2020, progressive disease was noted, prompting the initiation of 5-fluorouracil (5-FU) and oxaliplatin along with pembrolizumab. Since March 2020, pembrolizumab monotherapy was pursued with radiological evidence of excellent response. Pembrolizumab monotherapy was continued through August 2022, where positron emission tomography (PET)/CT scan showed no evidence of active disease. This case serves to supplement the ongoing evidence of ICI efficacy especially as a durable, sustained radiological response was evident for more than three years from the time of diagnosis.

## Introduction

Cholangiocarcinoma (CCA) originates from bile duct cells and accounts for 3% of all gastrointestinal tract tumors [1]. It is known for its poor prognosis [2]. Pembrolizumab is a monoclonal antibody directed against programmed cell death protein 1 (PD-1), which has been approved to treat cancers that have high microsatellite instability (MSI) and are mismatch repair deficient (MMRd). This has been used as a marker to predict a good response to the medication [3]. However, this is seen only in 2% of those with CCA [4]. Chemotherapy using gemcitabine and cisplatin has been recommended as the first line of management for those with advanced/unresectable CCA. The role of immune checkpoint inhibitors (ICIs) such as pembrolizumab is gaining more attention as evidence of its efficacy is emerging.

## Case presentation

**Figure 1:**
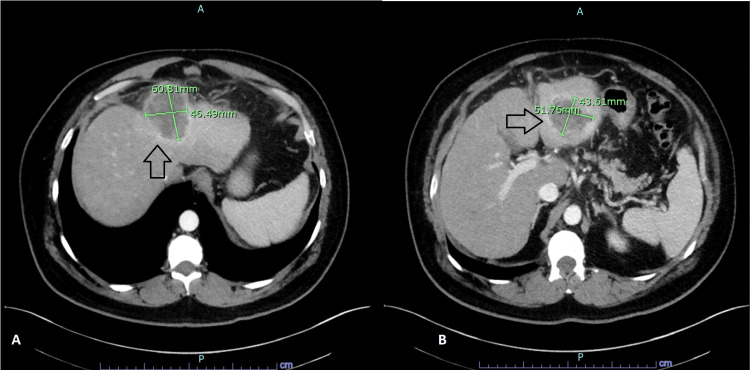
October 2019 CT scan of the abdomen There is evidence of two large left hepatic lobe masses (arrows in A and B)

In October 2019, a 52-year-old male patient presented to the emergency department (ED) with worsening abdominal pain for one month. The pain was described as right upper quadrant (RUQ) abdominal pain, intermittent, aching, aggravated by movement and oral intake, and occasionally radiating to the back with poor appetite and 35 pounds weight loss over the same period but no nausea, vomiting, and change in bowel habits. Past medical history was unremarkable except for diabetes type 2 on metformin. In the ED, vital signs included blood pressure of 140/80 mmHg, heart rate of 80 beats per minute, respiratory rate of 17 cycles per minute, temperature of 36.7 degrees Celsius, and peripheral oxygen saturation (SpO2) of 100 % in room air. On the physical exam, he had RUQ tenderness and moderate abdominal distention. Otherwise, the physical exam was unremarkable. Basic labs showed elevated alkaline phosphatase 313 U/L (reference 38-126), with normal aspartate aminotransferase (AST), alanine transaminase (ALT), and bilirubin. Otherwise, his labs were unremarkable. A CT scan of the abdomen and chest with IV contrast showed left paracardiac lymphadenopathy adjacent to a large 6 cm x 4 cm mass in the left hepatic lobe that was exophytic into the pericardial fat pad with another smaller 5 cm x 4 cm left hepatic lobe mass, one enlarged lymph node in the lower mediastinum measuring 3 cm, and multiple other enlarged lymph nodes affecting the porta hepatis region and left perigastric region (Figure 1). Alpha-fetoprotein (AFP) level was normal at 4.9 ng/ml (reference 0-8.3), and carcinoembryonic antigen (CEA) was within normal at 3.8 ng/ml (reference 0-4.7). Biopsy of the hepatic lesion revealed a malignant epithelioid neoplasm in the non-cirrhotic liver with immunoprofile that was compatible with stage 4 intrahepatic CCA (positive pan-cytokeratin (CK), CK7, 19, and 20, GATA3; and negative hepatocyte paraffin 1 (HepPar1), cancer antigen (CA)19.9, chromogranin, thyroid transcription factor (TTF)-1, NKX3.1 thrombomodulin, and uroplakin). Both esophagogastroduodenoscopy (EGD) and colonoscopy were unremarkable. The hepatitis panel was negative. He was started on cisplatin and gemcitabine FOLFOX chemotherapy.

**Figure 2:**
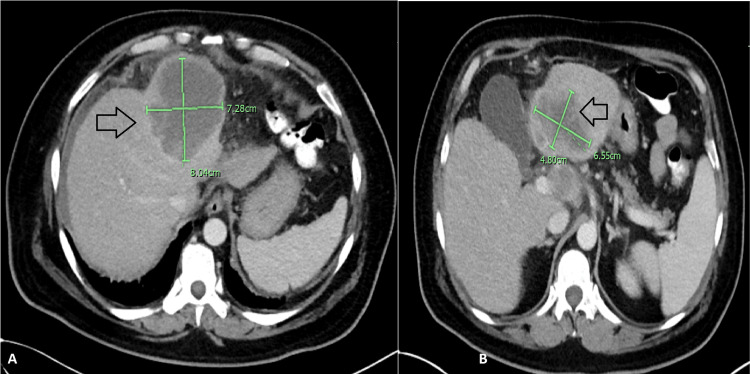
January 2020 CT scan of the abdomen Radiological evidence of progression in both left hepatic lobe masses is seen (arrows in A and B)

**Figure 3:**
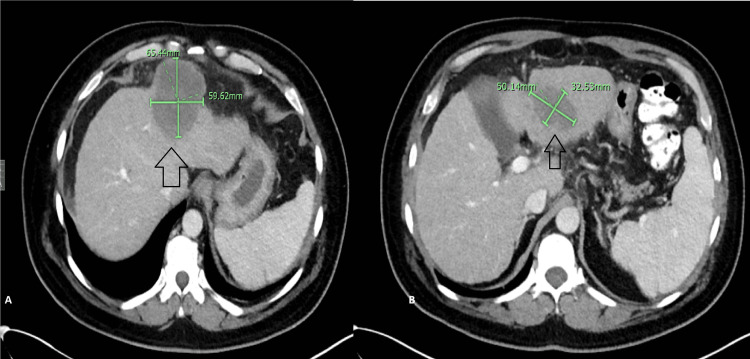
March 2020 CT scan of the abdomen Radiological evidence of response to 5-FU, oxaliplatin, and pembrolizumab with a relative reduction in the size of both left hepatic lobe masses is noted (arrows in A and B) 5-FU: 5-Fluorouracil

After his eighth cycle of chemotherapy, the patient was readmitted four months later (January 2020) with worsening RUQ abdominal pain for two weeks which was uncontrollable with opioids. A CT scan of the abdomen and chest showed evidence of progression despite being on chemotherapy (Figure 2). This prompted a change in his regimen to include 5-fluorouracil (5-FU), oxaliplatin, and leucovorin. Monthly pembrolizumab was also added to this regimen given high programmed cell death ligand 1 (PD-L1) expression of more than 90%, and negative Her2. This has shown dramatic pain control despite mild cytopenias including a platelet count of 128,000 cells/mcl and hemoglobin of 9.1 g/dl. After three cycles of oxaliplatin and 5-FU, in March 2020, a restaging CT scan showed an interval reduction in the size of both left hepatic masses to 4 cm x 2.4 cm and 2.6 cm x 1.8 cm with a resolution of periportal perigastric lymphadenopathy (LAP) and improving left para-aortic and mediastinal LAP (Figure 3). The patient remained on pembrolizumab alone, then it was discontinued as a result of worsening severe thrombocytopenia in December 2021. 

**Figure 4:**
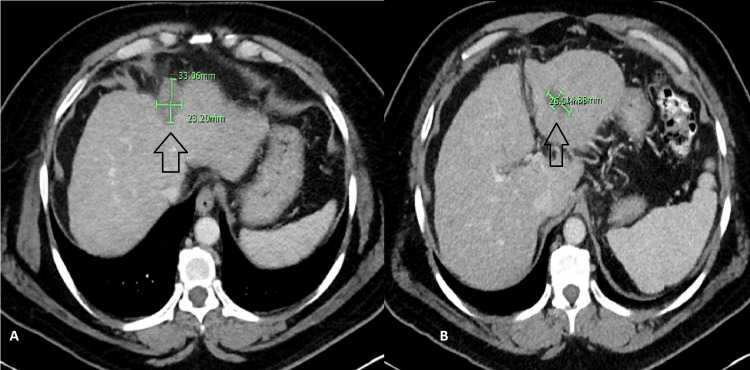
February 2021 CT scan of the abdomen Further evidence of the radiological response of the masses is observed (arrows in A and B)

**Figure 5:**
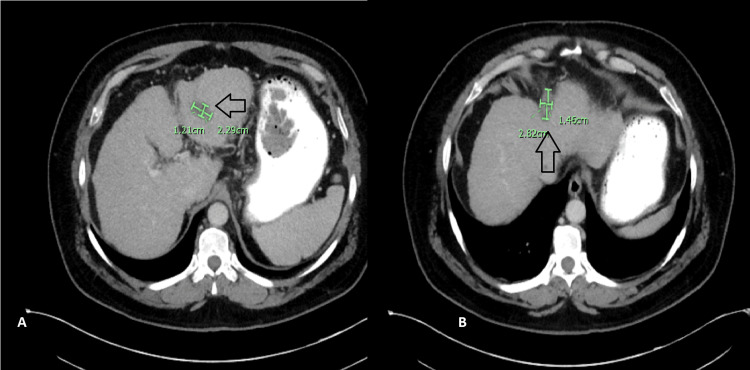
December 2021 CT scan of the abdomen A durable radiological response of the masses is seen (arrows in A and B)

**Figure 6:**
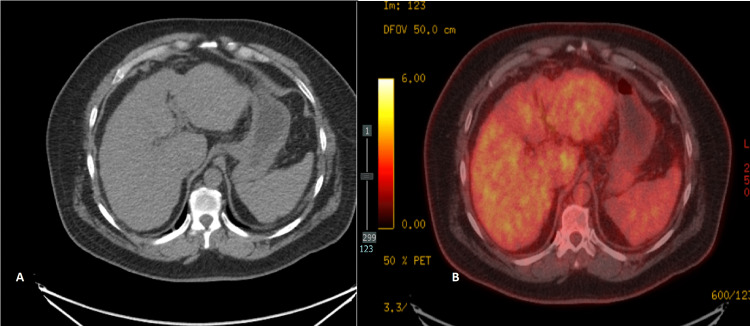
August 2022 PET/CT scan of the abdomen There was no radiological evidence of active hepatic disease in both A (CT scan) and B (PET scan) PET: Positron emission tomography

A repeat CT scan in February 2021 showed new development of cirrhosis but with further interval decrease in the size of left hepatic masses 3.3 cm x 2.3 cm and 2.6 cm x 1.4 cm (Figure 4). Pembrolizumab was resumed after the resolution of thrombocytopenia and repeat CT scans three (May 2021), seven (September 2021), and 10 (December 2021) months later showed stable left hepatic lobe lesions. The last (December 2021) CT scan also showed enlarging left para-aortic LAP with a further radiological response of the left hepatic lobe masses (Figure 5). The patient underwent CyberKnife (Accuray Inc., Sunnyvale, California, USA) image-guided stereotactic radiosurgery of the enlarging LAP in May 2022. Afterward, pembrolizumab was restarted and a positron emission tomography (PET)/CT scan in August 2022 showed stable hepatic lesions (Figure 6).

## Discussion

Classification of CCA is based on location and includes intrahepatic CCA and extrahepatic CCA. The latter can be further classified as perihilar and distant CCA [2]. Mortality and morbidity from CCA have recently risen and it is estimated that two-thirds of cases are unresectable [5]. For these patients, chemotherapy is recommended and according to the UK ABC-02 study the first-line chemotherapy is cisplatin and gemcitabine, which has an improved median overall survival of 11.7 months as compared to gemcitabine alone [2]. The efficacy of ICI monotherapy including pembrolizumab, nivolumab, and durvalumab in this setting is limited with response rates up to 13% in earlier studies [6]. Recently, the results from phase 3 of the placebo-controlled TOPAZ-1 trial supported chemoimmunotherapy (gemcitabine, cisplatin, and durvalumab) as first-line for advanced biliary tract cancers with improved median overall survival to 12.8 months [7]. Other ongoing trials to validate this suggestion include KEYNOTE-966 (pembrolizumab + cisplatin and gemcitabine), IMBRAVE 151 (atezolizumab + cisplatin and gemcitabine), M7824 (bintrafusp alpha + cisplatin and gemcitabine). 

Pembrolizumab has shown good response in certain malignancies including gastric, urothelial, non-small cell lung carcinoma, and melanoma [8-11]. Evidence of good response of advanced cholangiocarcinoma to pembrolizumab was provided by phase 2 of the KEYNOTE-158 study that enrolled 233 patients with non-colorectal cancer with MSI-high and MMRd [12]. This study included 22 patients with MSI-high CCA and showed an improved median overall survival of 24.3 months. This was better than the result in those with negative or unknown MSI status. The same result was seen in other studies of pembrolizumab monotherapy in advanced CCA with a median overall survival of 4.3 to 7.4 months in those with unknown or negative MSI status [13,4]. A common conclusion from these studies is that pembrolizumab has a better response among those with high MSI status as compared to unknown or negative MSI status, and when compared to the ABC-02 study, it had a more prolonged survival. 

Only a handful of cases of successful management of advanced CCA with pembrolizumab were reported in the literature (Table 1) [14-17]. All of these cases included advanced CCA with high MSI status that initially failed the recommended combined chemotherapy but achieved a good response to pembrolizumab. In addition, a recent case of good durable response to pembrolizumab and paclitaxel in advanced extrahepatic CCA with low MSI status was reported [18]. Interestingly in our case, a good durable response of the advanced CCA was achieved largely with pembrolizumab with a current survival rate of more than three years since diagnosis. The maintenance duration of ICIs remains debatable, but studies of ICIs in advanced biliary tumors were used for a duration of two years. However, there were no negative survival effects if ICIs were discontinued earlier [19].

**Table 1:** Previously reported cases with good response of CCA to pembrolizumab G: Gemcitabine; C: Cisplatin; S1: Combination of tegafur, gimeracil, and oteracil; MSI: Microsatellite instability, CCA: Cholangiocarcinoma

Reference	Age	Extent	Initial treatment	MSI status
Ikeda et al. [14]	50	Intrahepatic	G+C+S1 for 6 cycles	High
Czink et al. [15]	24	Extrahepatic	5-FU+Oxaliplatin+Panitumumab for 12 months followed by Irinotecan based therapy+Bevacizumab	High
Nakamura et al. [16]	69	Intrahepatic	Surgery+S1 for 4 months	High
Naganuma et al. [17]	60	Intrahepatic	Surgery+G+C+S1 for 1 year	High

## Conclusions

The role of ICIs in the management of patients with advanced unresectable biliary tract cancers including CCA is under continuous investigation. There is emerging evidence of efficacy and improved overall survival, prompting the recommendation of ICIs as first-line therapy in the appropriate setting. This case serves to supplement the ongoing evidence of this efficacy especially as durable sustained radiological response was evident for more than three years from the time of diagnosis.
